# Phytochemical Interactions with Calmodulin and Critical Calmodulin Binding Proteins Involved in Amyloidogenesis in Alzheimer’s Disease

**DOI:** 10.3390/biom13040678

**Published:** 2023-04-15

**Authors:** Danton H. O’Day

**Affiliations:** 1 Cell and Systems Biology, University of Toronto, Toronto, ON M5S 3G5, Canada; danton.oday@utoronto.ca; 2 Department of Biology, University of Toronto Mississauga, Mississauga, ON L5L 1C6, Canada

**Keywords:** calmodulin binding proteins, BACE1, Alzheimer’s disease, amyloid beta, amyloidogenesis, nutraceuticals, plant extracts, phytochemicals

## Abstract

An increasing number of plant-based herbal treatments, dietary supplements, medical foods and nutraceuticals and their component phytochemicals are used as alternative treatments to prevent or slow the onset and progression of Alzheimer’s disease. Their appeal stems from the fact that no current pharmaceutical or medical treatment can accomplish this. While a handful of pharmaceuticals are approved to treat Alzheimer’s, none has been shown to prevent, significantly slow or stop the disease. As a result, many see the appeal of alternative plant-based treatments as an option. Here, we show that many phytochemicals proposed or used as Alzheimer’s treatments share a common theme: they work via a calmodulin-mediated mode of action. Some phytochemicals bind to and inhibit calmodulin directly while others bind to and regulate calmodulin-binding proteins, including Aβ monomers and BACE1. Phytochemical binding to Aβ monomers can prevent the formation of Aβ oligomers. A limited number of phytochemicals are also known to stimulate calmodulin gene expression. The significance of these interactions to amyloidogenesis in Alzheimer’s disease is reviewed.

## 1. Introduction

Alzheimer’s disease (AD) is an internationally devastating disease on multiple levels: personal, social and financial [1]. With more than 55 million individuals dealing with the disease worldwide, the annual global cost of AD is estimated at USD 1.3 trillion. With a new case appearing every 3 s or so, 10 million more people will become AD sufferers every year. Hidden within these frightful numbers are not only those suffering from the disease but also the associated financial, temporal and mental stresses put on loved ones and caregivers. Thus, it is imperative that a cure, or at least a way of slowing disease progression, be found as quickly as possible.

**Figure 1:**
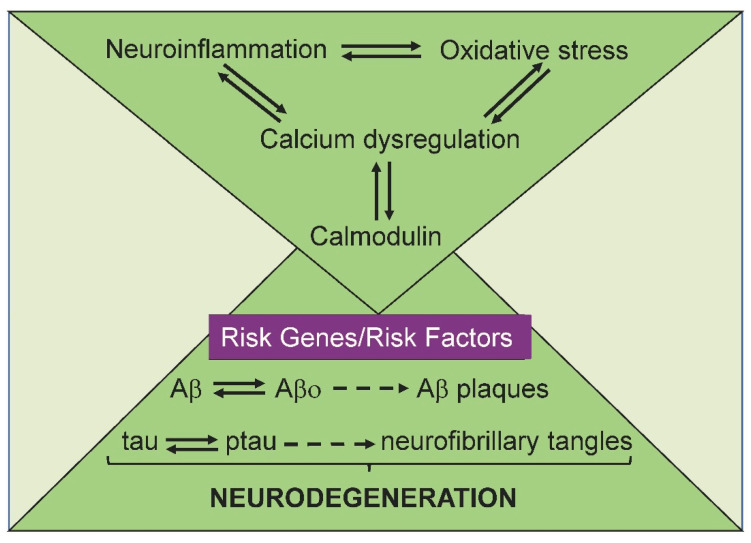
Some of the key events in onset and progression Alzheimer’s disease (AD). The interconnected early events of neuroinflammation, oxidative stress and calcium dysregulation lead to the activation of calmodulin. Together these events, and likely others, can interact with AD risk genes and risk factors leading to the formation of amyloid beta plaques and neurofibrillary tangles the classic hallmarks believed to underlie the resulting neurodegeneration that characterizes the disease. Aβ, amyloid beta; Aβo, amyloid beta oligomers; ptau, phosphorylated tau.

As summarized in Figure 1, areas of therapeutic focus include calcium dyshomeostasis, neuroinflammation, oxidative stress and the formation of the classic biomarkers (amyloid β (Aβ) oligomers and plaques, ptau and neurofibrillary tangles) all of which are inter-related to some degree [2,3,4]. Evidence exists that calcium dyshomeostasis activates calmodulin (CaM) that, in coordination with other factors, regulates risk proteins and genes involved in AD as discussed below [5,6,7]. The interplay between these events and their downstream effects are still being revealed and no AD therapies targeting them have been very successful [1,8,9]. AD disease progression is widely and historically attributed to the accumulation of extracellular amyloid beta (Aβ), oligomers (Aβo) and resulting senile plaques along with intracellular phosphorylated tau (p-tau) based neurofibrillary tangles [4].

Aβ is widely considered to be a critical player in AD pathogenesis, a role supported by its ability to cause synaptic dysfunction and neurodegeneration in vitro and in vivo [10]. Here the focus is on the amyloidogenic pathway and how phytochemicals may be useful as therapeutics. Aberrant, sequential processing of amyloid beta precursor protein (AβPP) by β-secretase and γ-secretase generates Aβ monomers that progressively oligomerize and aggregate with other components to form senile plaques [10]. Aβ40 and Aβ42 are major contributors to plaques with Ab42 being the more toxic form. AβPP can also be initially cleaved by α-secretase in a non-amyloidogenic pathway that fails to generate Aβ but instead produces non-toxic fragments [11,12]. However, pharmacological therapies targeting Aβ production, oligomerization and turnover have been unsuccessful in opening the door for immunotherapies with monoclonal antibodies, such as solanezumab, aducanumab and lecanemab that have recently been approved by the U.S. Food and Drug Administration [13]. As covered here, phytochemicals that target Aβ production and transformation into plaques offer an alternative and potentially beneficial way to tackle this problem.

There is accumulating evidence that some herbal medicines, nutraceuticals and supplements may provide alternative therapeutic options with reduced risk of side effects for a diversity of diseases, including Alzheimer’s [14,15,16,17,18]. Nutraceuticals or biopharmaceuticals are foods with proven or purported medical effects while herbal medicines are medicines specifically made from plants [16,19]. Herbal medicines and nutraceuticals have various biological effects due to their component phytochemicals. Most studies have been carried out using plant extracts that contain a diversity of constituents in various combinations, the types of phytochemicals and their concentrations further varying with the mode of extraction. Dietary supplements are natural or synthesized minerals, nutrients or other substances in a concentrated form that add a nutritional, health or physiological benefit [19]. Except for specific examples, in this review, the active ingredients of herbal treatments, dietary supplements, medical foods and other plant-based chemicals will simply be referred to as phytochemicals. Here, the primary focus is on specifically identified phytochemicals and their sources that not only have supporting evidence of therapeutic value in treating AD but also are involved in calmodulin-mediated events of amyloidogenesis.

## 2. Calmodulin and Alzheimer’s Disease

**Figure 2:**
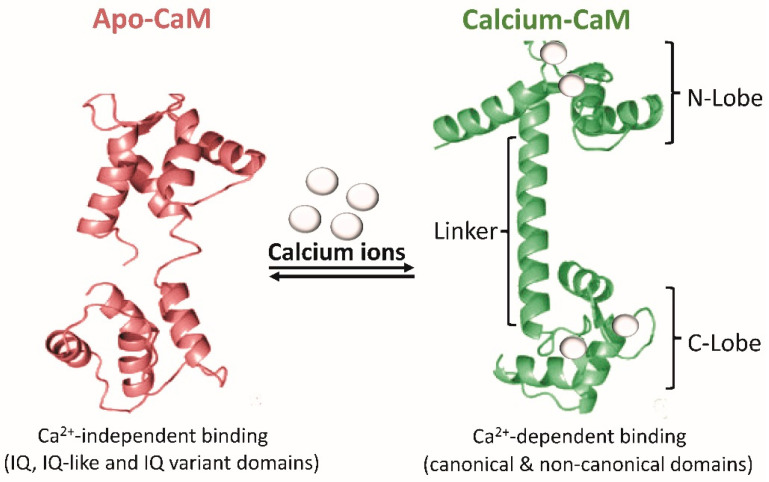
Conformational change in calmodulin (CaM) induced by calcium ions and its implications to target protein binding. These events are discussed, along with the various binding domains, in the main text.

A small (148aa; 16.7 kDa), highly conserved calcium-binding protein, calcium-free (apo-) or calcium-bound calmodulin (Ca^2+^/CaM) can bind to a diversity of CaM-binding proteins (CaMBPs) [20,21,22]. Two pairs of calcium-binding EF-hands, the N-terminal and C-terminal lobes are connected via a flexible linker (Figure 2). It is this binding domain flexibility that endows CaM with its ability to bind to and regulate such a diversity of proteins, many of which are involved in the onset and progression of AD [6,23]. In the presence of calcium ions, a conformational change exposes methionine-rich hydrophobic pockets that permit binding to target proteins via hydrophobic amino acid-rich CaM-binding domains (CaMBDs). Hydrophobic regions open up in each of CaM’s globular domains allowing target binding via a diversity of hydrophobic-rich CaMBDs. The flexible linker permits CaM to wrap around CaMBPs of various sizes. Unlike typical protein–protein binding, there are a number of canonical calcium-dependent CaMBD motifs as well as many non-canonical binding domains [20,21,22]. Calcium-dependent binding involves a wide assortment of canonical domain subclasses as well as more recently revealed non-canonical domains: ***1-10 Subclasses,*** 1-10, (FILVW)xxxxxxxx(FILVW)); 1-5-10, (FILVW)xxx(FAILVW)xxxx(FILVW); Basic 1-5-10, (RK)(RK)(RK)(FAILVW)xxx(FILV)xxxx(FILVW)); ***1-12 Subclass,*** 1-12, (FILVW)xxxxxxxxxxXXxx(FILVW); ***1-14 Subclasses,*** 1-14, (FILVW)xxxxxxxxxxxx(FILVW); 1-8-14, (FILVW)xxxxxx(FAILVW)xxxxx(FILVW); Basic1-8-14, (RK)(RK)(RK)(FILVW)xxxxxx(FAILVW)xxxxx(FILVW); 1-5-8-14, (FILVW)xxx(FAILVW)xx(FAILVW)xxxxx(FILVW); ***1-16 Subclass,*** 1-16, (FILVW)xxxxxxxxxxxxx(FILVW); ***Non-canonical,*** various hydrophobic aa arrangements; short sequences and myristoylated proteins. Over 300 calcium-dependent CaMBPs have been identified [21,22]. On the other hand, apo-CaM binds to a smaller population of calcium-independent CaMBPs via IQ [FILV]Qxxx[RK]Gxxx[RK]xx[FILVWY]; IQ-like [FILV]Qxxx[RK]Gxxxxxxxx); IQ-2A [IVL]QxxxRxxxx[VL][KR]xW; IQ-2B [IL]QxxCxxxxKxRxW and IQ variant [IVL]QxxxRxxxx[RK]xx[FILVWY] domains [20]. The chemical structure of CaM antagonists varies but routinely they are typically hydrophobic molecules with a net positive charge, as exemplified by calmidazolium binding, allowing them to bind to the hydrophobic patches and pockets of Ca^2+^/CaM thereby interfering with the binding and activation of target CaMBPs [23].

**Figure 3:**
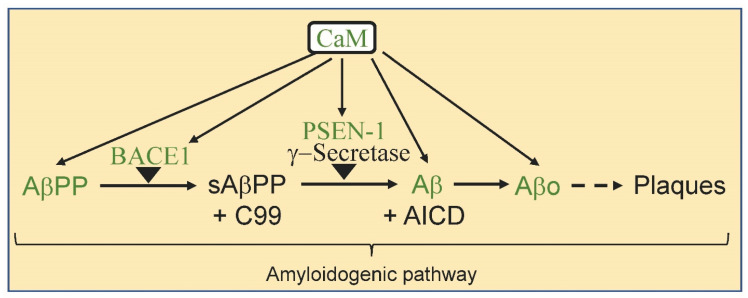
Calmodulin involvement in early events of amyloidogenic pathway. Aβ, amyloid beta; Aβo, amyloid beta oligomers; AβPP, amyloid-β precursor protein; sAβPP, amyloid-β precursor protein; AICD, APP intracellular domain; BACE1, beta-secretase 1; C99, 99-residue C-terminal fragment; CaM, calmodulin; PSEN-1, Presenilin-1.

Calmodulin is directly involved in binding to and controlling some of the major proteins involved in amyloidogenesis. It binds to and regulates beta-amyloid precursor protein enzyme 1 or beta-secretase (BACE1), the first enzyme in the amyloid pathway [6]. In the initial stages, it also binds to amyloid precursor protein (AβPP), the source of amyloid beta, and to PSEN-1, a component of γ-secretase the second enzyme that generates amyloid beta (Figure 3) [12]. Not only that CaM binds to amyloid beta (Aβ) itself, resulting in multiple feedback regulatory events [24,25].

CaM also has multiple other roles in AD. It regulates tau phosphorylation via two CaM-binding kinases CaMKII and cyclin-dependent kinase 5 (cdk5) and is a central regulatory element in synaptic events linked to learning and memory (e.g., CaMKII, PP2B) [26]. Finally, CaM binds to and regulates a number of CaMBPs involved in neuroinflammation and antioxidant functions, two events believed to be critical to the onset of AD as well as many other neurodegenerative diseases [27]. A large number of phytochemicals have been shown to have beneficial effects in the treatment of AD and there is accumulating evidence they may do so via their regulation of CaM and specific CaMBPs. Here, the focus is on phytochemicals and CaM-mediated early events of amyloidogenesis (Figure 3).

## 3. Calmodulin as a Phytochemical Target

Melatonin, quercetin and curcumin, three phytochemicals widely touted for their value in the treatment of AD, all bind to CaM [28,29,30]. A product of the pineal gland, melatonin (N-acetyl-5-methoxytryptamine) is a multifunctional hormone that regulates blood pressure, body temperature, circadian rhythm and sleep [31,32]. Melatonin has antioxidant, immunomodulatory and other activities that play a role in neurodegenerative diseases, such as Alzheimer’s. Recent research has revealed that at least part of these diverse and central functions is due to melatonin binding to CaM and its effects on BACE1 and other AD-related calmodulin-binding proteins. The preclinical and clinical research on melatonin that was carried out up to 2021 was reviewed but despite the detail, the melatonin’s impact on calcium and calmodulin signal transduction was overlooked [32]. 

In 1993, tritium-labeled melatonin was shown to bind to CaM [28]. Subsequently, the binding between melatonin and CaM was validated using a diversity of techniques and approaches (gel band shift assays, enzymatic competition assays with calcineurin, fluorescence spectroscopy, far and near UV circular dichroism, nuclear magnetic resonance (NMR) studies and by molecular dynamics simulations) [33,34]. The results reveal that the indolic ring of melatonin binds to the aliphatic amino acids in the hydrophobic cavities of each of the CaM globular domains. The lack of groups to interact with the acidic residues in the cavities means the binding of melatonin is weaker than other CaM antagonists, such as trifluoperazine (TFP) and W7 (N-(6-Aminohexyl)-5-chloro-1-naphthalenesulfonamide hydrochloride). Modifications of melatonin with appropriate NH2 side groups could increase the efficacy of CaM binding [33]. 

Melatonin also impacts calmodulin function in other ways. The post-translational modification of CaM, especially phosphorylation, affects its regulatory functions in the cell [35]. Depending on the residues that are phosphorylated, calcium-binding may be increased while the activity of target CaMBPs can be increased or inhibited by the phosphorylated CaM (pCaM). Melatonin is a neurohormone that can generate pCaM. At low concentrations, melatonin has been shown to cause the PKCα-mediated phosphorylation of serine and threonine residues in CaM in vitro and in vivo [36]. The authors speculate this could lead to the regulation of key enzymes during the circadian and sleep cycles. To date, no studies on the post-translational modification of CaM have been conducted for any neurodegenerative disease.

Quercetin has been shown to bind to CaM in a calcium-dependent manner and to inhibit tumor promoter-induced effects, such as the extensively studied CaM antagonist W7 (N-6-aminohexyl-5-chloro-1-naphthalenesufonamide) [28]. The hydroxyl group at C3 in quercetin appears to be central to BACE1 inhibition via the formation of hydrogen bonds that interfere with the catalytic center of the enzyme [37]. Overall, these data point to the direct (i.e., via BACE-1) and indirect (i.e., via CaM kinases) regulation of CaM signal transduction as part of quercetin’s main modes of action in AD.

The direct binding of a curcumin derivative to Ca^2+^/CaM has been demonstrated. Using a technique called “phage display biopanning”, Shin et al. [30] presented results showing that a curcumin derivative called HBC (4-[3,5-Bis-[2-(4-hydroxy-3-methoxy-phenyl)-ethyl]-4,5-dihydro-pyrazol-1-yl]-benzoic acid) directly interacts with Ca^2+^/CaM. The evidence revealed that HBC binds to the C-terminal hydrophobic pocket of Ca^2+^/CaM, the same site where the CaM antagonist W7 binds.

## 4. Amyloid Beta Monomers as a Phytochemical Targets

The aggregation of Aβ to form neurotoxic oligomers and fibrils is a key event in the generation of amyloid plaques, a central hallmark of AD and a potential cause of neurodegeneration. As a result, the inhibition of Aβ aggregation is an appealing approach for the treatment of AD. A number of phytochemicals have been shown to affect the amyloidogenic pathway with some showing direct binding to amyloid beta itself. There are three main ways phytochemicals have been shown to interfere with the aggregation of the Aβ peptide: (1) preventing the assembly of Aβ monomers into aggregates; (2) affecting the remodeling of Aβ oligomers; and (3) inhibiting secondary nucleation [38]. The methods and approaches for studying the events and mechanisms of Aβ aggregation and its inhibition have been reviewed [39].

Since Aβ is a CaMBP, the inhibition of its aggregation via direct phytochemical binding, which can involve both hydrogen bonding, hydrophobic and Van der Walls interactions, is of primary interest here because it can guide the development of therapeutic agents that could prevent aggregation without unwanted side effects. That said, only a few studies have detailed the molecular interaction of a phytochemical inhibitor and Aβ monomers. Isolated from the red maple (*Acer rubrum*), the phenolic compound ginnalin A, also known as acertannin, binds to Aβ dose-dependently inhibiting its aggregation and reversing fibrillogenesis [40]. Evidence revealed ginnalin A binds via residues 17–21, 35 and 38, residues implicated in β-sheet formation and fibril stabilization [41]. While ginnalin A binds directly to Aβ, it appears that other mechanisms mediate each of the events of aggregation, oligomer assembly and fibril disassembly.

Pagano et al. [39] list the names and chemical structure of 13 “natural products” that affect Aβ aggregation: brazilin, curcumin epigallocatechin gallate, ginnalin A, Wgx-50, myricetin, oleuropein, oleuropein aglycone, resveratrol, rosmarinic acid, sclerotionrin, tashinone and uncarinic acid C, not all of which bind directly to Aβ monomers. Brazilin binds to Aβ monomers, an event mediated by both hydrogen bonds and hydrophobic interactions via Aβ residues Leu17, Phe19 and Phe20 while fibrils preferentially interacted with salt-bridge residues Asp23-Lys28 [42].

NMR studies have revealed that curcumin, ferulic acid, myricetin and nordihydroguaiaretic acid all bind to Aβ monomers via the same amino acid regions: Arg5, Ser8, Gly9, His13, Lys16, Asp23 and Ile31 [43]. While the attention here has been on the binding of phytochemicals to Aβ, this binding can affect the subsequent fate of the potentially toxic peptide by impacting its aggregation and fibrillization. For example, the relative inhibitory activity of these phenolic compounds on the oligomerization of Aβ40 and Aβ42 shows the following inhibitory hierarchy: myricetin > nordihydroguaiaretic acid = ferulic acid ≥ curcumin [43]. Identifying the critical Aβ binding targets for phytochemicals can thus provide direction in the development of new analogs or variants with greater specificity in binding and inhibition of aggregation and fibrilization without unwanted side effects. Some phytochemical-Aβ binding occurs non-specifically. For example, epigallocatechin gallate, a component of the green tea binds with higher affinity to Aβ oligomers over monomers but the monomer binding is non-specific [44]. It should also be noted that the inhibition of Aβ aggregation and fibrilization can occur by other means that do not involve phytochemical binding to the monomer [43].

## 5. Bace1 as a Phytochemical Target

The structure and function of BACE1, mechanisms of inhibitor binding and its use as a therapeutic AD target have recently been reviewed [45,46]. Since BACE1 is the first enzyme in the amyloidogenic pathway it is in a primary position to prevent the formation of Aβ. However, the targeting of BACE1 as an AD therapy has been unsuccessful for a number of reasons. BACE1 is an aspartic protease that may cleave as many as 68 different substrates thus having important roles, including astrogenesis, axon growth, myelination, neurogenesis, neuronal migration and normal synaptic function, among others [47,48]. As a result, due to these many roles the complete removal of the enzyme using gene knockouts or the complete inhibition of enzyme activity has led to neurological and other defects [48,49]. On the other hand, significant but partial (50–80%) inhibition appears to avoid many of the abnormal effects, thus providing a new direction for targeting BACE1 in AD treatment [50,51]. Despite a long list of failed efforts, BACE1 remains a high-priority target for AD therapy. Phytochemicals could play a role in this fresh approach. Several studies have reported on BACE1 inhibiting phytochemicals that also bind to and inhibit acetylcholinesterase, another key target in AD [52,53,54]. There are arguments that such multi-directed agents may offer unique benefits over single target treatments but here we are specifically interested in what these phytochemicals can yield about the targeting BACE1 [55]. The goal here is to show that multiple phytochemicals bind to and inhibit the CaMBP BACE1 in support of the primary premise of this review that CaM and its targets are central to the onset and progression of AD and a diversity of phytochemicals exert their AD-related therapeutic effects via them.

Facing the necessity of crossing the blood–brain barrier (BBB), enzyme inhibitors used in the treatment of AD must be of low molecular weight and high lipophilicity adding to the value of many plant metabolites [56]. This concept is borne out by the large and diverse number of plant metabolites that not only can cross the BBB but also inhibit BACE1. In addition to binding directly to CaM and inducing its phosphorylation as discussed above, melatonin impacts CaM-mediated events of AD in another way: it inhibits BACE1 [45]. Five different melatonin derivatives inhibited the enzyme at levels of 75% or more at 5 μM. The different derivatives induced neurite growth and showed neuroprotective effects bound to different BACE1 residue combinations. BACE1 was first shown to possess a potential CaMBD indicating it was a putative CaMBP [5]. Subsequent in vitro studies revealed that BACE1 not only bound CaM in a calcium-dependent manner, this binding increased enzyme activity 2-fold [6]. The identified, but not experimentally validated, CaMBD was found within residues 65–75, a region with two independent binding motifs. Three melatonin variants (2, 3, 5) generated by Panyatip et al. [45] interact with Tyr71, suggesting they would impact CaM-binding to BACE1. These results support the authors’ contention that these melatonin derivatives could be developed as a specific therapeutic against BACE1 in the treatment of AD.

A diversity of flavonoids extracted from plants have also been shown to selectively inhibit BACE1 [18,37,46]. O-methylated quercetins isolated from *Caragana balchaschensis* (Kom.) Pojark are effective inhibitors of BACE1 revealing IC_50_ values from 1.2 to 6.5 μM [57]. Nobiletin (5,6,7,8,30,40-hexamethoxyflavone), tangeretin (5,6,7,8,40-pentamethoxyflavone) and sinensetin (5,6,7,30,40-pentamethoxyflavone), the most common polymehtoxyflavones (PMFs) found in citrus peel extracts, are inhibitors of BACE1 but not other selected serine proteases [58]. Each of these flavones showed significant, dose-dependent inhibition of BACE1 at micromolar levels. While each was strongly inhibitory, tangeretin was the most efficient BACE1 inhibitor (IC_50_, 4.9 × 10^−5^ M), nobiletin was second (IC_50_, 5.9 × 10^−5^ M) while sinensetin was least efficient (IC_50_, 6.3 × 10^−5^ M). Understanding the mode of interaction of inhibitors and their targets can guide research into the development of new inhibitory agents. Tangeretin forms hydrogen bonds with Ser10 and Thr232, nobiletin with Ala157, Val336 and Thr232 and sinensetin with Tyr71, Lys75, Trp76 and Tyr198 [58]. None of these amino acids are in the catalytic sites for BACE1. Other studies have shown the importance of Asp32 and Asp228 in BACE1 inhibition [59]. Yusof et al. [46] did an extensive analysis of 64 different flavones that act as BACE1 inhibitors, arriving at three main observations: (1) sugar moieties increase inhibition, (2) flavonoids contain 2 aromatic rings of which A is involved in hydrogen bonding while B uses van der Walls interaction, and (3) hydrogen bonding with catalytic site Asp32 and Asp228 is important. These insights should help in the development of new flavone derivatives with enhanced, specific BACE1 inhibition.

Members of the nutrient-rich cruciferous vegetable family Brassicaceae include Bok choy, broccoli, Brussels sprouts, cabbage, cauliflower, collard greens and kale among others. Their nutraceutical value comes in part from the presence of various carotenoids (beta-carotene, lutein, zeaxanthin), folate, isothiocyanates and several vitamins (C, E and K). Sulforaphane, an isothiocyanate found in cruciferous vegetables, has been shown to have antioxidant, anti-inflammatory and neuroprotective effects [60,61]. In mouse AD models, sulforaphane has been shown to exert neuroprotective effects from Aβ deposition and toxicity [62]. Subsequently, sulforaphane was shown to inhibit BACE1 in a non-competitive, dose-dependent manner (IC_50_ 2.80 × 10^−6^ M) [63]. In contrast to competitive inhibition of an enzyme’s active site, non-competitive inhibitor binding to allosteric sites can be used to selectively inhibit specific enzyme functions and potentially reduce side effects [64,65].

An ethyl acetate extract of rhizomes from *Smilax china* L. showed inhibition of BACE1, the first evidence for the BACE1-inhibiting ability of stilbenoids [66]. The identified compounds that acted non-competitively and were strongly inhibitory included a trans/cis-resveratrol mixture (IC_50_ 1.5 × 10^−5^ M), oxyresveratrol (IC_50_ 7.6 × 10^−6^ M), veraphenol (IC_50_ 4.2 × 10^−6^ M) and cis-scirpusin A (IC_50_ 1.0 × 10^−5^ M). Due to the presence of hydrophilic phenolic hydroxyl groups which impedes their movement across the BBB, these compounds may not have direct use as an AD therapeutic.

A 2018 study examining isoflavones as BACE1 inhibitors revealed that genistein acted as a reversible, non-competitive inhibitor forming hydrogen bonds with Asn37, Gln73 and Trp76 [67]. Good sources for genistein are legumes: fava bean (*Vicia faba*), kudzu (*Pueraria lobata*), lupine (*Lupinus spp*.), soybeans (*Glycine max*) and psoralea (*Psoralea corylifolia*). Green tea catechins, stilbenoids, coumarins, citrus flavonoids and ellagitannin have also been shown to act as non-competitive inhibitors of BACE1 [56,66,67,68]. 

The forest herb *Corydalis cava* (Fumariaceae) is a folk medicine used for the treatment of memory and other dysfunctions in AD. Identifying BACE1 inhibitors using a BACE1-Immobilized Enzyme Reactor followed by verification using Fluorescence Resonance Energy Transfer (FRET) assay, Chlebek et al. [69] discovered two alkaloids with strong BACE1 inhibitory activity: (−)-corycavamine and (+)-corynoline. Dose-dependent inhibition was observed at micromolar levels for the alkaloids that were able to pass the BBB. Contradictory results with different IC50 measurements suggest further study of these inhibitors is in order.

Cinnamon, a spice used for centuries as a traditional medicine, has a diversity of benefits for the treatment of AD including anti-inflammatory, antioxidant and immunomodulatory effects [70,71]. One bio-effective constituent is cinnamic acid, a BACE1 inhibitor. Sun et al. [72] have coupled cinnamic acid (*p*-hydroxy-cinnamic acid) with the phytochemical luteolin to develop BACE1 inhibitors with greater effectiveness. While each of them is a non-competitive inhibitor, they each bind to different regions of BACE1 [65]. A series of constructs resulted in a single flavone-cinnamic acid hybrid referred to as 7c (IC_50_ 1.44 × 10^−6^ M) that should be further examined as a potential therapy for AD [72]. This approach of developing phytochemical conjugates could possibly guide the discovery of BACE1 inhibitors that are specific for AβPP1 leading to reduced levels of Aβ, while leaving other essential substrates untouched. Coumarin, which has potential BACE1 inhibiting activity, is present in the cinnamon extract as well [72]. An aromatic organic chemical compound that is toxic in high concentrations, coumarin (1,2-benzopyrone) is found at low levels in cinnamon, bison and sweet grass, carrots, green tea and tonka beans. It has been reported to inhibit BACE1, but that inhibition appears to be non-specific since six dihydroxanthyletin-type coumarins from *Angelica decursiva* all inhibited acetylcholinesterase and butyrylcholinesterase as well [52]. The modes of binding and other results could provide useful insights to chemists in the design of specific BACE1 inhibitors.

Cinnamon and other phytochemicals have been used in aromatherapy as a treatment for AD and other health issues. Aromatherapy using essential oils from the true cinnamon tree (*Cinnamomum verum* J. Presl. (Lauraceae)), *Crocus sativus* L. (Iridaceae) and a handful of other plants for the treatment of memory loss is a traditional approach in Iranian medicine [73]. Some essential oils, with their low molecular weight and hydrophobicity, have been shown to have therapeutic value for the treatment of AD [74]. Of relevance here, essential oils from a Lavender plant species, *Lavandula luisieri*, efficiently inhibited BACE1 enzyme activity [75]. 

The potential value of targeting BACE1 with resveratrol and its derivatives continues to be studied. Initially, Marambaud et al. [76] presented data that resveratrol did not affect Aβ production and had no inhibitory effects on either BACE1 or γ-secretase. Two years later these results were contradicted when Jeon et al. [66] identified several resveratrol derivatives from the dried rhizomes of *Smilax china* L. that inhibited BACE1: a trans/cis-resveratrol mixture (IC_50_ 1.5 × 10^−5^ M), oxyresveratrol (IC_50_ 7.6 × 10^−6^ M), veraphenol (IC_50_ 4.2 × 10^−6^ M) and cis-scirpusin A (IC_50_ 1.0 × 10^−5^ M). They showed less inhibition of other serine proteases including chymotrypsin, trypsin, elastase and α-secretase. Subsequently, Choi et al. [77,78] showed that resveratrol and resveratrol oligomers had a dose-dependent, significant inhibitory effect on baculovirus-expressed BACE-1 in a FRET assay. Koukoulitsa et al. [79] developed four resveratrol analogs that were more effective inhibitors of BACE1 than resveratrol. These dual-action inhibitors also inhibited oxidative stress-induced neuronal cell death, but it is not clear if this was a secondary effect of BACE1 inhibition or another action of the analogs. Resveratrol is also used as a control in the analyses of agents that inhibit BACE1 [58]. 

## 6. Phytochemicals and Calmodulin Gene Expression

Another mode of phytochemical involvement in CaM signaling is through the regulation of CaM gene expression. Walnuts have antioxidant and anti-inflammatory activities in the brain potentially providing protection against the decline in cognitive function associated with neurodegeneration. Microglia are central agents linked to the harmful oxidative and inflammatory events underlying AD and other neurodegenerative diseases. Whole walnut extract (1.5, 3.0 or 6.0%) treatment of a rat microglial cell line causes a time and dose-dependent rise in intracellular calcium levels and CaM protein expression [80]. Lipopolysaccharide (LPS) treatment of cells and organisms can induce an acute inflammatory response, a model that is used to understand the events underlying it [81]. A component of Gram-negative bacterial cell walls, LPS triggers the release of inflammatory cytokines and activates Toll-like receptor 4 signal transduction. Research revealed that walnut can protect primary neuronal cells against calcium dysregulation induced by oxidative and inflammatory stress and can also suppress LPS-induced microglial activation [82]. Using an in vitro model, Thangthaeng et al. [80] demonstrated that walnut extract inhibits lipopolysaccharide (LPS)-induced microglial activation by inducing a rapid and sustainable rise in Ca^2+^ levels and stimulating the upregulation of CaM gene in a strain of highly aggressively proliferating immortalized microglial cells. CaM is essential for microglial activation where it has been shown to regulate microglial proliferation, ramification and phagocytic activity [83]. The discovery that walnut extracts can upregulate calcium levels and CaM expression in microglia provides evidence that bioactive compounds in walnuts can modulate microglial activation through CaM signaling. The specific bioactive components that cause these events and their mode(s) of action remain to be revealed.

A detailed, comprehensive review of the multitude of therapeutic benefits of both turmeric and curcumin has not only documented the worldwide uses of these agents but also delved into the cellular pathways they regulate [84]. Mainly found in the spice turmeric and curcumin, which binds to CaM and Aβ as detailed above, also acts as an acaricide, able to kill the carmine spider mite (*Tetranychus cinnabarinus*) apparently through the upregulation of the CaM gene, in turn, leading to the disruption of calcium homeostasis [85]. The induction of CaM gene expression by curcumin has not been studied in any other biological system.

## 7. Discussion

**Figure 4:**
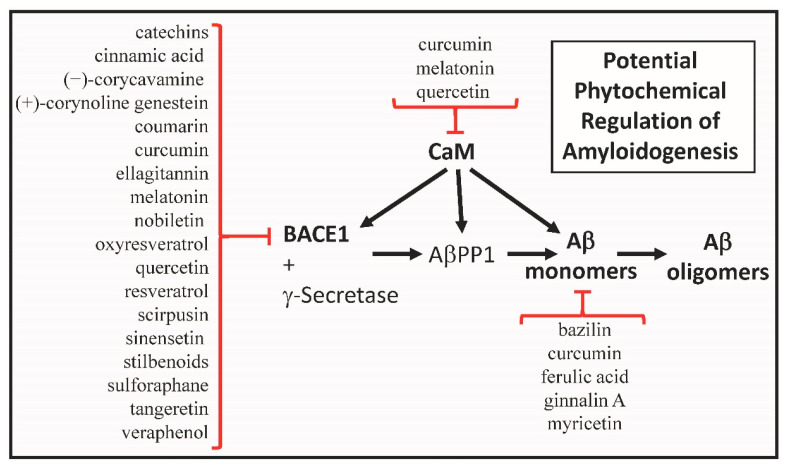
Phytochemical regulation of amyloidogenesis via calmodulin (CaM) and calmodulin binding proteins (bold).

There is increasing support for the use of some herbal medicines, nutraceuticals, plant extracts and supplements as alternative therapeutic options for the treatment of Alzheimer’s disease [17,18]. Numerous phytochemicals found within these alternative treatments fit the bill as potential AD therapeutics for a number of reasons [14,15,16,17,18]. First, they target specific, critical components (e.g., BACE1, Aβ). Second, they are small often with hydrophobic attributes allowing them to cross the blood–brain barrier, a major issue for many pharmaceuticals. Third, as natural compounds typically found in a diversity of widely used healthy foods, they are less likely to be toxic. Of interest here is evidence that many potentially useful phytochemicals for the treatment of AD operate through calmodulin-mediated signaling to regulate critical events in amyloidogenesis. Numerous phytochemicals operate directly or indirectly on intracellular calmodulin signaling by binding to CaM, or the CaMBPs BACE1 and Aβ (Figure 4).

Three well-studied phytochemicals—melatonin, quercetin and curcumin—have been shown to bind to and inhibit CaM. At least 18 phytochemicals, as well as many more of their variants, directly bind to and inhibit BACE1, the first enzyme in the amyloid pathway. Five others bind to Aβ interfering with its functions, including its transition to oligomeric forms. Curcumin and some unidentified phytochemical in walnut extract stimulate CaM gene expression. A single, early immunology study suggested CaM levels decrease in key brain regions in Alzheimer’s individuals [86]. There has been no follow up on this research. If this region-specific decrease can be validated, then restoring natural levels of CaM via phytochemical treatment should be evaluated further. Of more widespread interest, however, is the inhibition of BACE1 by phytochemicals, a research area that has seen intense, focus with the goal of finding a way to inhibit BACE1′s role in Aβ generation while not affecting its essential cellular functions.

The phytochemicals that act as specific inhibitors of BACE1 have effective IC50 values revealed from in vitro analyses. IC50 values, the chemical concentration at which 50% enzyme inhibition occurs, are critical for evaluating inhibitor effectiveness [87]. To date, while there are data for many compounds that effectively inhibit BACE1, there does not appear to be enough information to select a specific BACE1 inhibitor as the best choice. That said, O-methylated quercetins, sulforaphane and two resveratrol compounds (oxyresveratrol, veraphenol) appear to be worthy candidates for intense investigation and the further development of variant forms [57,63,67]. Without more in vivo data on these and other phytochemicals, this suggestion could be premature. Curcumin and quercetin are undergoing US-based clinical trials but only in relation to their roles in AD neuroinflammation [88].

In terms of CaM signaling, melatonin is unique because it impacts it in a number of ways. First, melatonin directly binds to CaM. As a CaMBP it also binds to BACE1, itself a CaMBP, possibly via BACE1′s CaM-binding domain. Finally, melatonin induces the phosphorylation of CaM by PKC, potentially affecting CaM signaling post-translationally. Quercetin is another phytochemical with diverse effects on calmodulin signaling since it binds calcium-dependently to CaM and also to BACE1. Unlike the majority of BACE1 inhibitors that have been studied, quercetin binds to the catalytic site of the enzyme.

Another appealing and potentially valuable area of research is preventing amyloid monomers from aggregating to form oligomers which ultimately progress to senile plaques. Here, we have examined the binding of phytochemicals to Aβ monomers, an event that can have multiple downstream results. Aβ is a CaMBP that also binds to dozens of Aβ receptors [24,25]. This interaction has multiple regulatory implications detailed elsewhere that might explain some of the problems with attempts to prevent the production of Aβ [24]. The binding of certain phytochemicals to Aβ monomers has also been shown to impact the transition from the monomeric to oligomeric form. Many well-studied phytochemicals bind to Aβ and affect its aggregation: brazilin, curcumin, ferulic acid, ginnalin A, myricetin and others (Figure 2) [39,42,43]. These and other studies have also revealed the primary binding regions for these phytochemicals providing an introductory roadmap on how to find and develop new and more effective phytochemical-based drugs to stop Aβ aggregation. While it was beyond the scope of this review, a number of recent reviews have focused on research on the phytochemicals covered here outlining the type of study, organisms used and the resulting AD pathophysiological benefits [32,89,90,91,92,93,94].

## 8. Conclusions

An extensive amount of research continues to support the Calmodulin Hypothesis for AD [5,6,24]. Here, we show that many phytochemicals with real therapeutic potential either bind to CaM itself, CaMBPs (Aβ, BACE1) or affect CaM gene expression. Having revealed a number of purified and characterized phytochemicals with specific effects on critical CaM-mediated events involved in amyloidogenesis it seems the time is right to move on from using crude biological extracts with dozens to hundreds of component compounds some of which may be more harmful than beneficial. A primary question is: “Should researchers keep analyzing complex mixtures of plant extracts for new drugs or is there enough targets already on which to focus?” By spreading research funding too thinly we keep adding imprecise information while not moving ahead as quickly as is required to tackle one of the most devastating diseases facing humankind.

## Data Availability

Not applicable.

## Abbreviations

Aβ	amyloid beta
Aβo	amyloid beta oligomers
AβPP	amyloid-β precursor protein
AchR	acetylcholine receptor
AD	Alzheimer’s disease
AICD	APP intracellular domain
BACE1	beta-secretase 1
C99	99-residue C-terminal fragment
CaM	calmodulin
CaMBD	calmodulin binding domain
CaMBP	calmodulin binding protein
CaMKII	calcium/CaM-dependent kinase II
cdk5	cyclin dependent kinase 5
HBC	4-[3,5-Bis-[2-(4-hydroxy-3-methoxy-phenyl)-ethyl]-4,5-dihydro-pyrazol-1-yl]-benzoic acid)
NMR	nuclear magnetic resonance
PMFs	polymehtoxyflavones
PP2B	calcineurin
pTau	phosphorylated Tau
W7	N-6-aminohexyl-5-chloro-1-naphthalenesufonamide

## References

[1] Gauthier S., Webster C., Servaes S., Morais J.A., Rosa-Neto P. (2022). World Alzheimer Report 2022: Life after Diagnosis: Navigating Treatment, Care and Support.

[2] Khachaturian Z.S., Kay D.S., Burrows G.W. (1984). Towards theories of brain aging. Handbook of Studies on Psychiatry and Old Age.

[3] Hampel H., Caraci F., Cuello A.C., Caruso G., Nisticò R., Corbo M., Baldacci1 F., Toschi1 N., Garaci F., Chiesa P.A. (2020). A path toward precision medicine for neuroinflammatory mechanisms in Alzheimer’s disease. Front. Immunol..

[4] Karen E., De Strooper B. (2022). The amyloid hypothesis in Alzheimer disease: New insights from new therapeutics. Nature.

[5] O’Day D.H., Myre M.A. (2004). Calmodulin-binding domains in Alzheimer’s disease proteins: Extending the calcium hypothesis. Biochem. Biophys. Res. Commun..

[6] O’Day D.H., Eshak K., Myre M.A. (2015). Calmodulin Binding Proteins and Alzheimer’s Disease: A Review. J. Alz. Dis..

[7] O’Day D.H. (2020). Calmodulin binding proteins and Alzheimer’s disease: Biomarkers, regulatory enzymes and receptors that are regulated by calmodulin. Int. J. Mol. Sci..

[8] French J.A., Koepp M., Naegelin Y., Vigevano F., Rho J.M., Rosenberg E., Devinsky O., Olofsson P.S., Dichter M.A. (2017). Clinical studies and anti-inflammatory mechanisms of treatments. Epilepsia.

[9] Teleanu D.M., Niculescu A.-G., Lungu I.I., Radu C.I., Vladâcenco O., Roza E., Costăchescu B., Grumezescu A.M., Teleanu R.I. (2022). An overview of oxidative stress, neuroinflammation, and neurodegenerative diseases. Int. J. Mol. Sci..

[10] Selkoe D.J., Hardy J. (2016). The amyloid hypothesis of Alzheimer’s disease at 25 years. EMBO Mol. Med..

[11] Sahlin C., Pettersson F.E., Nilsson L.N., Lannfelt L., Johansson A.S. (2007). Docosahexaenoic acid stimulates non-amyloidogenic APP processing resulting in reduced Abeta levels in cellular models of Alzheimer’s disease. Eur. J. Neurosci..

[12] Canobbio I., Catricalà S., Balduini C., Torti M. (2011). Calmodulin regulates the non-amyloidogenic metabolism of amyloid precursor protein in platelets. Biochim. Biophys. Acta Bioenergy.

[13] Song C., Shi J., Zhang P., Zhang Y., Xu J., Zhao L., Zhang R., Wang H., Chen H. (2022). Immunotherapy for Alzheimer’s disease: Targeting β-amyloid and beyond. Trans. Neurodegen..

[14] Farías G.A., Guzmán-Martínez L., Delgado C., Maccioni R.B. (2014). Nutraceuticals: A novel concept in the prevention and treatment of Alzheimer’s disease and related disorders. J. Alz. Dis..

[15] Jantan I., Ahmad W., Bukhari S.N.A. (2015). Plant-derived immunomodulators: An insight on their preclinical evaluation and clinical trials. Front. Plant Sci..

[16] AlAli M., Alqubaisy M., Aljaafari M.N., AlAli A.O., Baqais L., Molouki A., Abushelaibi A., Lai K.-S., Lim S.-H.E. (2021). Nutraceuticals: Transformation of conventional foods into health promoters/disease preventers and safety considerations. Molecules.

[17] Azlan U.K., Annuar N.A.K., Mediani A., Aizat W.M., Damanhuri H.A., Tong X., Yanagisawa D., Tooyama I., Ngah W.Z.W., Jantan I. (2022). An insight into the neuroprotective and anti-neuroinflammatory effects and mechanisms of *Moringa oleifera*. Front. Pharmacol..

[18] Maccioni R.B., Calfío C., González A., Lüttges V. (2022). Novel Nutraceutical Compounds in Alzheimer Prevention. Biomolecules.

[19] Santini A., Novellino E. (2017). To Nutraceuticals and back: Rethinking a concept. Foods.

[20] Rhoads A.R., Friedberg F. (1997). Sequence motifs for calmodulin recognition. FASEB J..

[21] Tidow H., Nissen P. (2013). Structural diversity of calmodulin binding to its target sites. FEBS J..

[22] Grant B.M.M., Enomoto M., Ikura M., Marshall C.B. (2020). A non-canonical calmodulin target motif comprising a polybasic region and lapidated terminal residue regulates localization. Int. J. Mol. Sci..

[23] Léger C., Pitard I., Sadi M., Carvalho N., Brier S., Mechaly A., Raoux-Barbot D., Davi M., Hoos S., Weber P. (2022). Patrice Vachette Dynamics and structural changes of calmodulin upon interaction with the antagonist calmidazolium. BMC Biol..

[24] O’Day D.H. (2023). Calmodulin and amyloid beta as coregulators of critical events during the onset and progression of Alzheimer’s disease. Int. J. Mol. Sci..

[25] Poejo J., Salazar J., Mata A.M., Gutierrez-Merino C. (2021). The relevance of amyloid β-calmodulin complexation in neurons and brain degeneration in Alzheimer’s disease. Int. J. Mol. Sci..

[26] Ghosh A., Geise K.P. (2015). Calcium/calmodulin-dependent kinase II and Alzheimer’s disease. Molec. Brain.

[27] O’Day D.H. (2022). Calmodulin binding domains in critical risk proteins involved in neurodegeneration. Curr. Issues Mol. Biol..

[28] Nishino H., Naito E., Iwashima A., Tanaka K.I., Matsuura T., Fujiki H., Sugimura T. (1984). Interaction between quercetin and Ca^2+^-calmodulin complex: Possible mechanism for anti-tumor-promoting action of the flavonoid. GANN Jpn. J. Cancer Res..

[29] Benítez-King G., Huerto-Delgadillo L., Antón-Tay F. (1993). Binding of 3H-melatonin to calmodulin. Life Sci..

[30] Shin J.S., Lee J., Park H.J., Park S.J., Kwon H.J. (2004). A new curcumin derivative, HBC, interferes with the cell cycle progression of colon cancer cells via antagonization of the Ca^2+^/calmodulin function. Chem Biol..

[31] Lerner A.B., Case J.D. (1960). Melatonin. Fed. Proc..

[32] Roy J., Wong K.Y., Aquili L., Uddin M.S., Heng B.C., Tipoe G.L., Wong K.H., Fung M.L., Lim L.W. (2022). Role of melatonin in Alzheimer’s disease: From preclinical studies to melatonin-based therapies. Front. Neuroendocrinol..

[33] Ouyang H., Vogel H.J. (1998). Melatonin and serotonin interactions with calmodulin: NMR, spectroscopic and biochemical studies. Biochim. Biophys. Acta Protein Struct. Mol. Enzymol..

[34] Turjanski A.G., Estrin D.A., Rosenstein R.E., McCormick J.E., Martin S.R., Pastore A., Biekofsky R.R., Martorana V. (2004). NMR and Molecular Dynamics Studies of the Interaction of Melatonin with Calmodulin. Protein Sci..

[35] Villalobo A. (2018). The multifunctional role of phosphor-calmodulin in pathophysiological processes. Biochem. J..

[36] Soto-Vega E., Meza I., Ramirez-Rodriguez G., Benitez-King G. (2004). Melatonin stimulates calmodulin phosphorylation by protein kinase C. J. Pineal Res..

[37] Shimmyo Y., Kihara T., Akaike A., Niidome T., Sugimoto H. (2008). Flavonols and flavones as BACE-1 inhibitors: Structure–activity relationship in cell-free, cell-based and in silico studies reveal novel pharmacophore features. Biochim. Biophys. Acta.

[38] Piccialli I., Tedeschi V., Caputo L., D’Errico S., Ciccone R., De Feo V., Secondo A., Pannaccione A. (2020). Exploring the therapeutic potential of phytochemicals in Alzheimer’s. disease: Focus on polyphenols and monoterpenes. Front. Pharmacol..

[39] Pagano K., Tomaselli S., Molinari H., Ragona L. (2020). Natural Compounds as Inhibitors of Ab Peptide Aggregation: Chemical Requirements and Molecular Mechanisms. Front. Neurosci..

[40] Fan Q., Liu Y., Wang X., Zhang Z., Fu Y., Liu L., Wang P., Ma H., Ma H., Seeram N.P. (2020). Ginnalin A Inhibits Aggregation, Reverses Fibrillogenesis, and Alleviates Cytotoxicity of Amyloid β(1-42). ACS Chem. Neurosci..

[41] Ahmed M., Davis J., Aucoin D., Sato T., Ahuja S., Aimoto S., Elliott J.I., Van Nostrand W.E., Smith S.O. (2010). Structural conversion of neurotoxic amyloid-beta (1-42) oligomers to fibrils. Nat. Struct. Mol. Biol..

[42] Du W.J., Guo J.J., Gao M.T., Hu S.Q., Dong X.Y., Han Y.F., Liu F.F., Jiang S., Sun Y. (2015). Brazilin inhibits amyloid beta-protein fibrillogenesis, remodels amyloid fibrils and reduces amyloid cytotoxicity. Sci. Rep..

[43] Ono K., Li L., Takamura Y., Yoshiike Y., Zhu L., Han F., Mao X., Ikeda T., Takasaki J.-I., Nishijo H. (2012). Phenolic compounds prevent amyloid beta-protein oligomerization and synaptic dysfunction by site-specific binding. J. Biol. Chem..

[44] Ahmed R., VanSchouwen B., Jafari N., Ni X., Ortega J., Melacini G. (2017). Molecular mechanism for the (-)-epigallocatechin gallate-induced toxic to nontoxic remodeling of abeta oligomers. J. Am. Chem. Soc..

[45] Panyatip P., Tadtong S., Sousa E., Puthongking P. (2020). BACE1 inhibitor, neuroprotective, and neuritogenic activities of melatonin derivatives. Sci. Pharm..

[46] Yusof M.N.I.S., Abdullah Z.L., Othman N., Fauzi F.M. (2022). Structure–activity relationship analysis of flavonoids and its inhibitory activity against BACE1 enzyme toward a better therapy for Alzheimer’s disease. Front. Chem..

[47] Hemming M.L., Elias J.E., Gygi S.P., Selkoe D.J. (2009). Identification of beta-secretase (BACE1) substrates using quantitative proteomics. PLoS ONE.

[48] Yan R. (2017). Physiological functions of the b-site amyloid precursor protein cleaving enzyme 1 and 2. Front. Mol. Neurosci..

[49] McConlogue L., Buttini M., Anderson J.P., Brigham E.F., Chen K.S., Freedman S.B., Games D., Johnson-Wood K., Lee M., Zeller M. (2007). Partial reduction of BACE1 has dramatic effects on Alzheimer plaque and synaptic pathology in APP Transgenic Mice. J. Biol. Chem..

[50] Kimura R., Devi L., Ohno M. (2010). Partial reduction of BACE1 improves synaptic plasticity, recent and remote memories in Alzheimer’s disease transgenic mice. J. Neurochem..

[51] Barão S., Moechars D., Lichtenthaler S.F., De Strooper B. (2016). BACE1 Physiological Functions May Limit Its Use as Therapeutic Target for Alzheimer’s Disease. Trends Neurosci..

[52] Ali M.Y., Seong S.H., Jung H.A., Jannat S., Choi J.S. (2018). Kinetics and molecular docking of dihydroxanthyletin-type coumarins from *Angelica decursiva* that inhibit cholinesterases and BACE1. Arch. Pharm. Res..

[53] Janat S., Balupuri A., Ali M.Y., Hong S.S., Choi C.W., Choi Y.-H., Ku J.-M., Kim W.J., Leem J.Y., Kim J.E. (2019). Inhibition of β-site amyloid precursor protein cleaving enzyme 1 and cholinesterases by pterosins via a specific structure-activity relationship with a strong BBB permeability. Exp. Mol. Med..

[54] Saeedi M., Iraji A., Vahedi-Mazdabadi Y., Alizadeh A., Edraki N., Firuzi O., Eftekhari M., Akbarzadeh T. (2022). *Cinnamomum verum* J. Presl. bark essential oil: In vitro investigation of anti-cholinesterase, anti-BACE1, and neuroprotective activity. BMC Complement. Med. Therap..

[55] Sharifi-Rad J., Rapposelli S., Sestito S., Herrera-Bravo J., Arancibia-Diaz A., Salazar L.A., Yeskaliyeva B., Beyatli A., Leyva-Gómez G., González-Contreras C. (2022). Multi-target mechanisms of phytochemicals in Alzheimer’s disease: Effects on oxidative stress, neuroinflammation and protein aggregation. J. Pers. Med..

[56] Jeon S.Y., Bae K., Seong Y.H., Song K.S. (2003). Green tea catechins as a BACE1 (beta-secretase) inhibitor. Bioorg. Med. Chem. Lett..

[57] Zhumanova K., Lee G., Baiseitova A., Shah A.B., Kim J.H., Kim J.Y., Lee K.W., Park K.H. (2021). Inhibitory mechanism of O-methylated quercetins, highly potent β-secretase inhibitors isolated from *Caragana balchaschensis* (Kom.) Pojark. J. Ethnopharmacol..

[58] Youn K., Yu Y., Lee J., Jeong W.-S., Ho C.-T., Jun M. (2017). Polymethoxyflavones: Novel β-secretase (BACE1) inhibitors from citrus peels. Nutrients.

[59] Hernández-Rodríguez M., Correa-Basurto J., Gutiérrez A., Vitorica J., Rosales-Hernández M.C. (2016). Asp32 and Asp228 Determine the Selective Inhibition of BACE1 as Shown by Docking and Molecular Dynamics Simulations. Eur. J. Med. Chem..

[60] Tarozzi A., Angeloni C., Malaguti M., Morroni F., Hrelia S., Hrelia P. (2013). Sulforaphane as a potential protective phytochemical against neurodegenerative diseases. Oxid. Med. Cell. Longev..

[61] Brandenburg L.O., Kipp M., Lucius R., Pufe T., Wruck C.J. (2010). Sulforaphane suppresses LPS-induced inflammation in primary rat microglia. Inflamm. Res..

[62] Zhang R., Miao Q.W., Zhu C.X., Zhao Y., Liu L., Yang J., Li A. (2015). Sulforaphane ameliorates neurobehavioral deficits and protects the brain from amyloid beta deposits and peroxidation in mice with Alzheimer-like lesions. Am. J. Alzheimer’s Dis. Other Dement..

[63] Youn K., Yoon J.-H., Lee N., Lim G., Lee J., Sang S., Ho C.-T., Jun M. (2020). Discovery of sulforaphane as a potent BACE1 inhibitor based on kinetics and computational studies. Nutrients.

[64] Williams P., Sorribas A., Howes M.J. (2010). Natural products as a source of Alzheimer’s drug leads. Nat. Prod. Rep..

[65] Fang W.-S., Sun D.-Y., Yang S., Cheng C., Moschke K., Li T., Sun S., Lichtenthaler S.F., Huang J., Wang Y. (2019). Discovery of a series of selective and cell permeable beta-secretase (BACE1) inhibitors by fragment linking with the assistance of STD-NMR. Bioorg. Chem..

[66] Jeon S.Y., Kwon S.H., Seong Y.H., Bae K., Hur J.M., Lee Y.-Y., Suh D.-Y., Song K.-S. (2007). b-Secretase (BACE1)-inhibiting stilbenoids from Smilax Rhizoma. Phytomedicine.

[67] Youn K., Park J., Lee S., Lee S., Lee J., Yun E., Jeong W.-S., Jun M. (2018). BACE1 Inhibition by genistein: Biological evaluation, kinetic analysis, and molecular docking simulation. J. Med. Food.

[68] Ali M.Y., Jannat S., Jung H.A., Choi R.J., Roy A., Choi J.S. (2016). Anti-Alzheimer’s disease potential of coumarins from *Angelica decursiva* and *Artemisia capillaris* and structure-activity analysis. Asian Pac. J. Trop. Med..

[69] Chlebek J., De Simone A., Hošťálková A., Opletal L., Pérez C., Pérez D.I., Havlíková L., Cahlíková L., Andrisano V. (2016). Application of BACE1 immobilized enzyme reactor for the characterization of multifunctional alkaloids from *Corydalis cava* (Fumariaceae) as Alzheimer’s disease targets. Fitoterapia.

[70] Gruenwald J., Freder J., Armbruester N. (2010). Cinnamon and health. Crit. Rev. Food Sci. Nutr..

[71] Qian D., Wang Q., Lin S., Li Y., Gu X., Xia C., Xu Y., Zhang T., Yang L., Wu Q. (2022). Identification of potential targets of cinnamon for treatment against Alzheimer’s disease-related GABA-ergic synaptic dysfunction using network pharmacology. Sci. Rep..

[72] Sun D.-Y., Cheng C., Moschke K., Huang J., Fang W.-S. (2020). Extensive-structure modification on luteolin-cinnamic acid conjugates leading to BACE1 inhibitors with optimal pharmacological properties. Molecules.

[73] Khan A.H. (2009). The Greatest Elixir (Exir Azam).

[74] Benny A., Thomas J. (2019). Essential oils as treatment strategy for Alzheimer’s disease: Current and future perspectives. Planta Med..

[75] Videira R., Castanheira P., Grãos M., Salgueiro L., Faro C., Cavaleiro C. (2013). A necrodane monoterpenoid from Lavandula luisieri essential oil as a cell-permeable inhibitor of BACE-1, the β-secretase in Alzheimer’s disease. Flavour Fragr. J..

[76] Marambaud P., Zhao H., Davies P. (2005). Resveratrol promotes clearance of Alzheimer’s disease amyloid-b peptides. J. Biol. Chem..

[77] Choi C.W., Choi Y.H., Cha M.-R., Kim Y.S., Yon G.H., Hong K.S., Park W.-K., Kim Y.H., Ryu S.Y. (2011). In vitro BACE-1 inhibitory activity of resveratrol oligomers from the seed extract of *Paeonia lactiflora*. Planta Med..

[78] Choi Y.H., Yoo M.Y., Choi C.W., Cha M.R., Yon G.H., Kwon D.Y., Kim Y.S., Park W.-K., Ryu S.Y. (2009). A new specific BACE-1 inhibitor from the stem bark extract of *Vitis vinifera*. Planta Med..

[79] Koukoulitsa C., Villalonga-Barber C., Csonka R., Alexi X., Leonis G., Dellis D., Hamelink E., Belda O., Barry R., Micha-Screttas M. (2016). Biological and computational evaluation of resveratrol inhibitors against Alzheimer’s disease. J. Enzyme Inhib. Med. Chem..

[80] Thangthaeng M. (2018). Walnut extract modulates activation of microglia through alteration in intracellular calcium concentration. Nutrition Res..

[81] Ngkelo A., Meja K., Yeadon M., Adcock I., Kirkham P.A. (2012). LPS induced inflammatory responses in human peripheral blood mononuclear cells is mediated through NOX4 and Giα dependent PI-3 kinase signalling. J. Inflamm..

[82] Carey A.N., Fisher D.R., Joseph J.A., Shukitt-Hale B. (2013). The ability of walnut extract and fatty acids to protect against the deleterious effects of oxidative stress and inflammation in hippocampal cells. Nutr. Neurosci..

[83] Casal C., Tusell J.M., Serratosa J. (2001). Role of calmodulin in the differentiation/activation of microglial cells. Brain Res..

[84] Fuloria S., Mehta J., Chandel A., Sekar M., Rani N.N.I.M., Begum M.Y., Subramaniyan V., Chidambaram K., Thangavelu L., Nordin R. (2022). A comprehensive review on the therapeutic potential of *Curcuma longa* Linn. in relation to its major active constituent curcumin. Front. Pharmacol..

[85] Zhou H., Guo F., Luo J., Zhang Y., Liu J., Zhang Y., Zheng X., Wan F., Ding W. (2021). Functional analysis of an up regulated calmodulin gene related to the agaricidal activity of curcumin against *Tetranychus cinnabarinus* (Boisduval). Pest Management Sci..

[86] McLachlan D.R.C., Wong L., Bergeron C., Baimbridge K.G. (1987). Calmodulin and Calbindin D28K in Alzheimer Disease. Alzheimer Dis. Assoc. Disord..

[87] Caldwell G.W., Yan Z., Lang W., Masucci J.A. (2012). The IC(50) concept revisited. Curr. Top. Med. Chem..

[88] Cummings J., Lee G., Nahed P., Kambar M.E.Z.N., Zhong K., Fonseca J., Taghva K. (2022). Alzheimer’s disease drug development pipeline: 2022. Alzheimer’s Dement..

[89] Alaqeel N.K., AlSheikh M.H., Al-Hariri M.T. (2022). Quercetin nanoemulsion ameliorates neuronal dysfunction in experimental Alzheimer’s disease model. Antioxidants.

[90] Campisi A., Sposito G., Pellitteri R., Santonocito D., Bisicchia J., Raciti G., Russo C., Nardiello P., Pignatello R., Casamenti F. (2022). Effect of unloaded and curcumin-loaded solid lipid nanoparticles on tissue transglutaminase isoforms expression levels in an experimental model of Alzheimer’s disease. Antioxidants.

[91] Gregory J., Vengalasetti Y.V., Bredesen D.E., Rao R.V. (2021). Neuroprotective Herbs for the Management of Alzheimer’s Disease. Biomolecules.

[92] Khan H., Ullah H., Aschner M., Cheang W.S., Akkol E.K. (2020). Neuroprotective effects of quercetin in Alzheimer’s disease. Biomolecules.

[93] Singh S., Kola P., Kaur D., Singla G., Mishra V., Panesar P.S., Mallikarjunan K., Krishania M. (2021). Therapeutic potential of nutraceuticals and dietary supplements in the prevention of viral diseases: A review. Front. Nutr..

[94] Zhou X., Venigalla M., Raju R., Münch G. (2022). Pharmacological considerations for treating neuroinflammation with curcumin in Alzheimer’s disease. J. Neural Trans..

